# A preliminary study on the assessment of bone age in children and adolescents in frigid-zone regions by the CHN-05 method

**DOI:** 10.3389/fmed.2026.1962869

**Published:** 2026-09-30

**Authors:** Yue Qi, Xinyi Li, Dongmei Lv, Xiaoyue Zhang, Jiawen Luo, Deli Zhao, Jinling Zhang

**Affiliations:** 1 Department of Radiology, Heilongjiang Hospital of Beijing Children’s Hospital, Capital Medical University(Harbin Children’s Hospital), Harbin, China; 2 Department of CT, ZhengZhou Central Hospital, Henan, China; 3 Department of CT, The Second Affiliated Hospital of Harbin Medical University, Harbin, China; 4 Department of CT, Harbin The Frist Hospital, Harbin, China; 5 Department of CT, The Sixth Affiliated Hospital of Harbin Medical University, Harbin, China

**Keywords:** bone age, CHN-05 method, chronological age, growth and development assessment, x-ray

## Abstract

**Background:**

Bone age assessment is a key tool for monitoring growth and development in children and adolescents. This study analyzes bone age development characteristics among children and adolescents aged 6–18 years, to provide reference data for improving regional bone age standards.

**Methods:**

Bone age data were retrospectively collected and assessed using the CHN-05 method. The correlation and differences between bone age and chronological age were analyzed, developmental patterns were compared by gender, and regression equations were established.

**Results:**

The CHN-05 method showed a strong correlation between bone age and chronological age (R = 0.952, *p* < 0.001). A higher proportion of females had advanced bone age compared to males, whereas a higher proportion of males had delayed bone age. In males, mean bone age was slightly lower than chronological age before age 14 and slightly higher after 14, with significant differences at ages 7, 8, and 12 (*p* < 0.05). In females, mean bone age was slightly higher before age 15 and slightly lower after 15, No statistically significant differences were observed among individual age strata. (*p* > 0.05). Gender-specific linear regression equations were established: males: y = 0.886x + 1.445 (R = 0.96, *p* < 0.001); females: y = 0.893x + 1.082 (R = 0.95, *p* < 0.001).

**Conclusion:**

The CHN-05 method is reasonably suitable for bone age assessment in children and adolescents and provides valuable reference data for refining regional bone age standards.

## Introduction

Bone age assessment provides a more accurate reflection of children's biological age and helps monitor their growth and development (1, 2). Today, bone age assessment is widely used in various key areas, including, but not limited to, the accurate prediction of adolescent height, the scientific selection of sports specialties, and the development of early detection and prevention strategies for a range of diseases (3–5).

In the context of the long-term trend of accelerated growth and development among Chinese children, the State General Administration of Sport promulgated the “*the Standards of Skeletal Age in Hand and Wrist for Chinese -China 05* ”, also known as the CHN-05 method (6). This method was developed based on the TW3 method, with adaptations for different application purposes, among which the RUS-CHN method (7) is the primary recommended approach for a more detailed evaluation of bone development maturity. Key factors influencing bone development and maturation include endocrine hormones, genetics, nutrition, and chronic diseases (8). Additionally, geographic environment, climatic conditions, dietary habits, as well as socio-economic and educational levels, significantly impact the growth and development of children ^(^9). Therefore, a comprehensive evaluation of bone development in children and adolescents across various regions is necessary. Given the lack of previous studies on the bone age of children and adolescents in frigid-zone regions, this study employs the CHN-05 method to retrospectively analyze the bone age of children and adolescents aged 6–18 years. The aim is to provide a preliminary understanding of the development of children and adolescents, and to establish a reference for regional bone age standards.

## Materials and methods

This study is a multicenter study. Clinical data and hand-wrist radiographic images of children and adolescents aged 6–18 years who underwent examinations for routine physical assessment or height prediction between March 2023 and December 2023 were retrospectively collected and analysed from Heilongjiang Hospital of Beijing Children's Hospital, the Second Affiliated Hospital of Harbin Medical University and the Sixth Affiliated Hospital of Harbin Medical University.

Harbin is the northernmost provincial capital city in China (45.7° N, 126.6° E; urban altitude ranging from 132 m to 200 m, with a mean altitude of 151 m). It has winter conditions for nearly half of the year with long-lasting snow cover and low annual mean temperature, representing a typical cold-climate region. All participants in the present study were long-term residents of this area.

A total of 1,527 cases were included, comprising 767 males and 760 females. The inclusion criteria were as follows: (1) moderate nutritional status and a history of good health. (2) height and weight within the normal range (mean ± 2 SD) for their age group. The exclusion criteria were: (1) the presence of conditions that impair growth and development, including tuberculosis, endocrine disorders, chronic wasting diseases, heart disease, etc. (2) use of medications that affect bone development, such as hormones. (3) individuals with significant psychological disorders, depression, or other mental illnesses and (4) individuals with evident traumatic injuries to the wrist. The study received approval from the hospital's ethics committee and was conducted in accordance with the Declaration of Helsinki. Written informed consent was obtained from the guardians of all participants. To fully protect participant privacy, clinical data has been anonymized by replacing names with identification numbers. The data for this study became accessible on December 31, 2023. During or after the data collection period, information that could potentially identify individual participants may be accessible.

### X-ray method

A posterior-anterior radiographic image of the left wrist was obtained using a mobile digital x-ray DR imaging system (Rekor DR7500). The subject remained in either a standing or sitting position, with the arm extended forward and placed on the examination table. Both the upper arm, forearm, and hand were fully straightened. During imaging, the forearm, palm, and wrist were aligned in the same plane as closely as possible. Simultaneously, the middle finger, third metacarpal bone, and the forearm axis were positioned in a straight line. The palm was placed flat and pressed tightly against the table surface, with the five fingers naturally separated. The thumb and index finger formed an approximate 30° angle. The center of the x-ray tube was aligned with the third metacarpophalangeal joint gap, and the x-ray was directed vertically to cover the entire metacarpophalangeal and carpal bones, as well as the distal ulna and radius, at a distance of 3.0 to 4.0 cm. The focal length was 75–85 cm, the exposure time was 0.1 s, the tube voltage was 45–50 kV, and the tube current was 300 mA.

### Bone age assessment methods and bone development determination criteria

Bone age assessment using the CHN-05 method (RUS-CHN): Based on the grading criteria outlined in the “Chinese Children and Youth Wrist Bone Maturity and Evaluation Method” (hereinafter referred to as the CHN-05 method), the Xigao-Tech Children and Youth Growth and Development Artificial Intelligence Evaluation AI System automatically reads the bone grades and calculates the corresponding bone age.

Determination criteria (10): A bone age and chronological age difference (BA-CA) within 1 year is classified as “synchronized development.” A BA-CA ≥ 1 year is classified as “developmental advancement,” and a BA-CA ≤ −1 year is classified as “developmental delay.” Potential disease risk is suggested when BA-CA is ≥ 2 years or ≤ −2 years.

### Image interpretation consistency test

A total of 60 hand-wrist radiographs were selected by stratified random sampling. Manual bone age assessment was performed by one radiologist who had received standardized CHN-05 training. The agreement between CHN-05 AI and manual assessment was evaluated using the intraclass correlation coefficient (ICC). Pearson correlation coefficient was used to analyze the correlation between the two methods. Systematic error was represented by the mean difference of bone age, and random error was expressed by the 95% confidence interval (95% CI) of the differences.

### Statistical methods

Statistical analyses were performed using SPSS 26.0 and R 4.5.0 software. Bone age values were treated as continuous variables, and results expressed as mean ± standard deviation (SD). The Pearson correlation method was used to analyze the relationship between bone age and chronological age. The intraclass correlation coefficient (ICC) with 95% confidence interval was calculated using a two-way random-effects model to evaluate the agreement between bone age and chronological age, followed by Bland-Altman analysis. Chi-square test or Fisher's exact test was employed to compare physical development between different genders. Multiple-comparison paired t-tests were applied to compare bone age with chronological age, with *P*-values adjusted by the Benjamini-Hochberg (FDR) method. Linear regression analysis was conducted to establish a regression model for the relationship between bone age and chronological age. A *p*-value of <0.05 was considered statistically significant.

## Result

### Patient information

A total of 1,527 children and adolescents were enrolled in this study, including 767 males and 760 females. The mean chronological age was 11.39 ± 2.92 years for males and 11.02 ± 2.81 years for females. The results are presented in Table 1.

**Table 1:** Basic information on height and weight by age group.

Groups	Male				Female			
	n	Age (years)	Height（cm）	Weight（kg）	n	Age (years)	Height（cm）	Weight（kg）
6.0∼	42	6.54 ± 0.25	119.56 ± 3.61	22.50 ± 2.37	51	6.45 ± 0.29	119.22 ± 3.92	22.63 ± 2.58
7.0∼	57	7.36 ± 0.32	124.95 ± 4.22	24.87 ± 3.23	69	7.41 ± 0.29	125.98 ± 4.19	24.62 ± 3.03
8.0∼	62	8.52 ± 0.29	131.97 ± 4.38	28.96 ± 4.17	80	8.49 ± 0.28	132.40 ± 4.89	28.14 ± 3.98
9.0∼	62	9.44 ± 0.31	136.50 ± 4.25	33.16 ± 4.81	99	9.44 ± 0.30	138.21 ± 5.03	31.75 ± 4.64
10.0∼	87	10.47 ± 0.30	142.54 ± 4.81	38.88 ± 5.68	97	10.41 ± 0.29	144.92 ± 5.46	36.48 ± 6.07
11.0∼	90	11.39 ± 0.30	148.99 ± 5.72	45.13 ± 6.67	79	11.43 ± 0.30	149.77 ± 4.86	40.89 ± 5.86
12.0∼	78	12.39 ± 0.29	155.76 ± 6.33	47.04 ± 8.47	71	12.42 ± 0.31	154.41 ± 4.76	44.48 ± 6.53
13.0∼	94	13.42 ± 0.30	162.25 ± 6.30	53.11 ± 8.10	72	13.41 ± 0.28	158.22 ± 4.38	47.92 ± 5.64
14.0∼	64	14.38 ± 0.25	167.70 ± 4.95	59.67 ± 8.03	60	14.37 ± 0.25	159.23 ± 4.96	49.65 ± 5.17
15.0∼	54	15.44 ± 0.28	171.26 ± 5.40	64.19 ± 7.52	48	15.49 ± 0.31	160.28 ± 4.58	49.03 ± 5.83
16.0∼	58	16.46 ± 0.31	172.30 ± 4.55	64.58 ± 5.67	31	16.33 ± 0.24	162.06 ± 4.05	50.75 ± 4.16
17.0∼	19	17.38 ± 0.29	173.64 ± 4.21	67.85 ± 7.61	3	17.00 ± 0.00	163.33 ± 3.79	52.77 ± 3.45

Consistency analysis showed that the mean difference in bone age between CHN-05 AI and manual assessment was 0.040 (95% CI: −0.0314–0.1114, *p* = 0.267). A high correlation was observed between the two methods (r = 0.993, *p* < 0.001), and the intraclass correlation coefficient (ICC) was 0.993 (95% CI: 0.989–0.996, *p* < 0.001).

### Analysis of the correlation between bone age and chronological age

Pearson’s correlation analysis revealed a significant positive correlation between bone age and chronological age in 1,527 children and adolescents: overall r = 0.952 (*p* < 0.01), for males (r = 0.956, *p* < 0.01), and for females r = 0.948 (*p* < 0.01).

ICC analysis based on a two-way random-effects model revealed excellent agreement between bone age and chronological age among 1,527 children and adolescents in Harbin. The single-measurement ICC was 0.950 (95%CI: 0.945–0.955), and the average-measurement ICC was 0.974 (95%CI: 0.972–0.977). In males, the single-measurement ICC was 0.953 (95%CI: 0.946–0.959), and the average-measurement ICC was 0.976 (95%CI: 0.972–0.979). In females, the single-measurement ICC was 0.946 (95%CI: 0.937–0.953), and the average-measurement ICC was 0.972 (95%CI: 0.968–0.976).(The Bland-Altman plot are shown in Figure 1).

**Figure 1:**
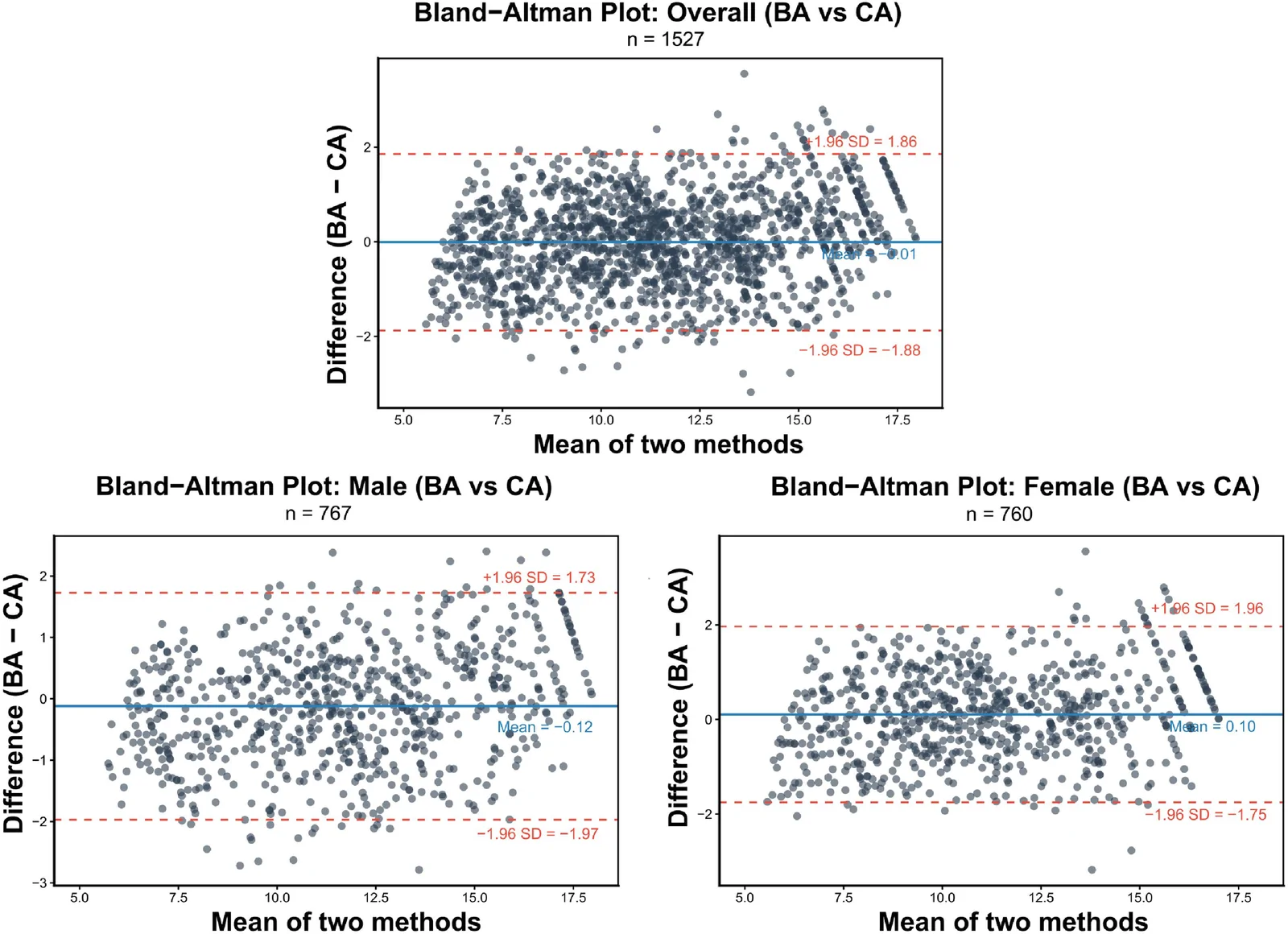
Bland-Altman plot of the differences between bone age and chronological age.

### Analysis of physical development of adolescents of different genders

Among the children and adolescents, the incidence of advanced development (1 ≤ BA-CA < 2) was higher in females (16.05%) than in males (10.95%) (*p* < 0.05). The incidence of advanced development with a risk of potential disease (BA-CA ≥ 2) was higher in females (2.11%) than in males (0.65%) (*p* < 0.05). The incidence of delayed development (−2 < BA-CA ≤ −1) was higher in males (16.30%) than in females (12.50%) (*p* < 0.05). The incidence of delayed development with a risk of potential disease (BA-CA ≤ −2) was higher in males (2.09%) than in females (0.39%) (*p* < 0.05) (Table 2).

**Table 2:** Analysis of physical development of children and adolescents of different genders [n (%)].

Developmental type	Male(*n* = 767)	Female(*n* = 760)	*χ^2^*	*p*
BA-CA ≥ 2	5 (0.65)	16 (2.11)	4.922	0.027
1 ≤ BA-CA < 2	84 (10.95)	122 (16.05)	8.079	0.004
−1 <BA-CA < 1	537 (70.01)	524 (68.95)	0.157	0.692
−2 <BA-CA ≤ −1	125 (16.30)	95 (12.50)	4.161	0.041
BA-CA ≤ −2	16 (2.09)	3 (0.39)	-	0.004

In the sample of 1,527 cases, the maximum developmental advance in males was 2.4 years (Figure 2a), and the maximum developmental delay was 2.8 years (Figure 2b). In females, the greatest developmental advance was 3.6 years (Figure 2c), and the greatest developmental delay was 3.2 years (Figure 2d).

**Figure 2:**
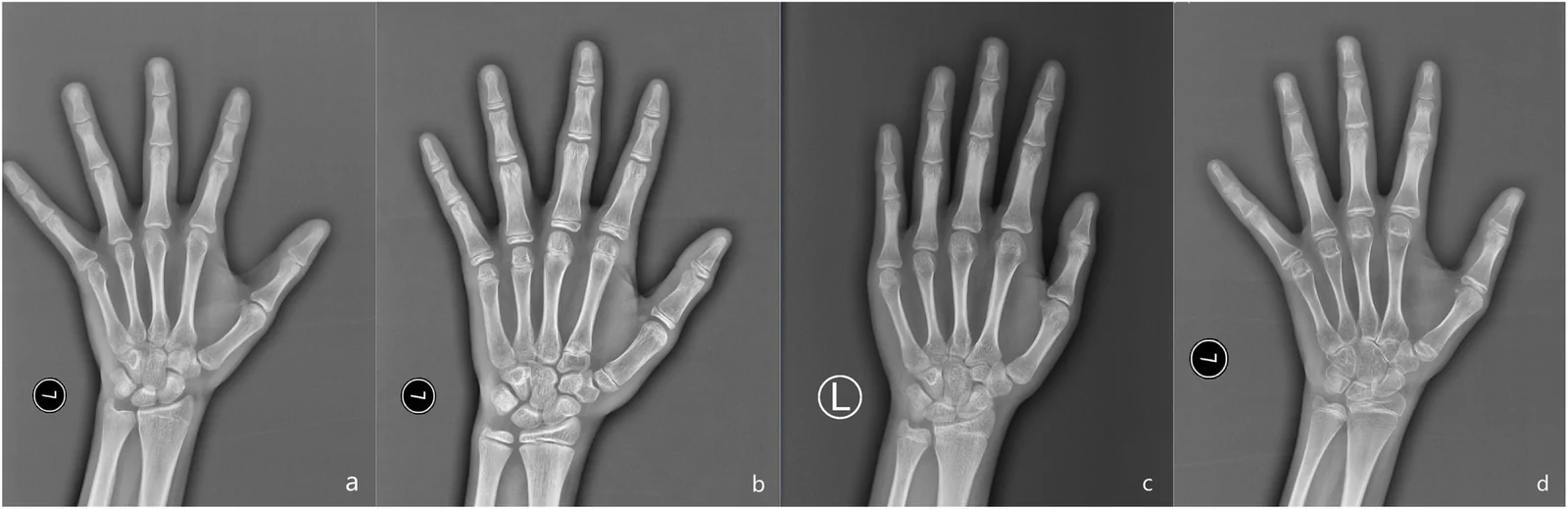
Bone age hand-wrist radiographs.

### Comparison of differences in bone age and chronological age

In the male group, the mean bone age was slightly lower than chronological age before 14 years, and slightly higher after 14 years. The bone age of the 6, 9, 11, and 14-year-old groups was close to the chronological age, while the 7, 8, and 12-year-old groups were significantly different from chronological age (*p* < 0.05). In the female group, the mean bone age was slightly higher than chronological age in the groups aged 15 years and younger, and slightly lower after 15 years. The bone age of the 6, 8, and 11-year-old groups was close to chronological age, with no significant differences observed among age groups. The results are shown in Table 3.

**Table 3:** Differential analysis of bone age and chronological age in the CHN-05 method.

Sex	Groups	N	Chronological age (years)	Bone age (years)	Difference (years)	*Raw p*	*FDR-adjusted p*
Male	6.0∼	42	6.54 ± 0.25	6.47 ± 0.73	−0.07 ± 0.72	0.552	0.736
	7.0∼	57	7.38 ± 0.29	7.10 ± 0.64	−0.27 ± 0.67*	0.003	0.026
	8.0∼	62	8.52 ± 0.29	8.05 ± 1.03	−0.47 ± 0.99*	<0.001	0.006
	9.0∼	62	9.45 ± 0.29	9.47 ± 0.86	0.02 ± 0.84	0.852	0.852
	10.0∼	87	10.47 ± 0.30	10.27 ± 1.01	−0.20 ± 1.05	0.085	0.338
	11.0∼	90	11.39 ± 0.30	11.37 ± 0.87	−0.03 ± 0.86	0.773	0.852
	12.0∼	78	12.39 ± 0.29	12.03 ± 0.91	−0.35 ± 0.86*	<0.001	0.006
	13.0∼	94	13.42 ± 0.30	13.19 ± 1.07	−0.23 ± 1.03	0.032	0.168
	14.0∼	64	14.38 ± 0.25	14.42 ± 1.00	0.03 ± 0.94	0.791	0.852
	15.0∼	54	15.44 ± 0.28	15.62 ± 1.18	0.17 ± 1.15	0.269	0.546
	16.0∼	58	16.46 ± 0.31	16.60 ± 1.06	0.14 ± 1.00	0.273	0.546
	17.0∼	19	17.38 ± 0.29	17.50 ± 0.54	0.12 ± 0.59	0.405	0.648
Female	6.0∼	51	6.45 ± 0.29	6.42 ± 0.82	−0.04 ± 0.87	0.763	0.852
	7.0∼	69	7.41 ± 0.29	7.51 ± 0.98	0.11 ± 0.95	0.360	0.617
	8.0∼	80	8.49 ± 0.28	8.51 ± 0.97	0.02 ± 0.99	0.841	0.852
	9.0∼	99	9.44 ± 0.30	9.58 ± 0.88	0.14 ± 0.88	0.116	0.374
	10.0∼	97	10.41 ± 0.29	10.59 ± 0.83	0.17 ± 0.80	0.035	0.168
	11.0∼	79	11.43 ± 0.30	11.48 ± 0.96	0.05 ± 0.91	0.629	0.794
	12.0∼	71	12.42 ± 0.31	12.54 ± 1.02	0.12 ± 0.98	0.298	0.551
	13.0∼	72	13.41 ± 0.28	13.56 ± 0.98	0.15 ± 0.90	0.160	0.426
	14.0∼	60	14.37 ± 0.25	14.56 ± 1.23	0.19 ± 1.21	0.226	0.542
	15.0∼	48	15.49 ± 0.31	15.75 ± 1.18	0.25 ± 1.13	0.125	0.374
	16.0∼	31	16.33 ± 0.24	16.20 ± 0.96	−0.13 ± 0.93	0.459	0.648
	17.0∼	3	16.99 ± 0.02	16.53 ± 0.81	−0.46 ± 0.82	0.436	0.648

### Linear regression analysis of bone age and chronological age

A strong linear regression relationship was observed between bone age and chronological age using the CHN-05 method. The regression equations for estimating chronological age based on bone age were derived from linear regression analysis: for males, y = 0.886x + 1.445, with a correlation coefficient of R = 0.96 (*p* < 0.001), for females, y = 0.893x + 1.082, with a correlation coefficient of R = 0.95 (*p* < 0.001). All regressions were highly significant.

## Discussion

Skeletal development exhibits continuity and occurs in stages, with skeletal morphology varying across different ages. This variation allows for the determination of chronological age, as well as the growth and developmental potential of children and adolescents (11). The growth and development patterns of children and adolescents in different regions are influenced by various factors, including geographic and climatic conditions, dietary habits, and socio-economic and educational levels. China is a vast and diverse country, with numerous ethnic groups and significant natural environmental variations, including marked climatic differences between the northern and southern regions. Therefore, the findings of this study may provide valuable insights for economically developed frigid-zone regions.

In this study, the CHN-05 method was used to assess the bone age development of children and adolescents aged 6–18 years. The results revealed a strong correlation between bone age values measured using the CHN-05 method and chronological age, with a significant positive linear relationship. These findings suggest that the CHN-05 method effectively reflects the trend of bone age development in children and adolescents, and the results are reliable.

This study identified gender differences in adolescent bone age by comparing the bone age of males and females. Specifically, the proportion of females exhibiting developmental advancement was higher than that of males, while the proportion of males showing developmental delay was higher than that of females. These differences were statistically significant (*p* < 0.05). These findings are consistent with those reported in previous studies by domestic scholars (12, 13). Bone age reflects the developmental stage of osteogenesis within the epiphysis and growth plate cartilage, with hormones, particularly estrogen, playing a crucial role in the process of endochondral ossification. Estrogen is essential for the fusion and closure of the growth plate in both sexes. Because estrogen levels are higher in females than in males before puberty, skeletal maturation occurs earlier in females at all ages. The earlier skeletal maturation observed in females prior to puberty is thought to be estrogen-driven, with elevated estrogen levels promoting linear growth, bone maturation, and epiphyseal closure (14), which contributes to sex differences in adolescent bone age.

In the male group, the mean values of bone age at the age of 6, 9, 11 and 14 are basically close to the chronological age, while the 7, 8, and 12-year-old groups were significantly different from chronological age (*p* < 0.05); the mean values of bone age before the age of 14 are slightly lower than the chronological age, and after the age of 14 are slightly higher than the chronological age. This indicates that males have a slightly delayed trend in pre-puberty and puberty (before 14 years old), and a catching up trend in late puberty (after 14 years old), which slightly exceeds the living age. Similar to the findings reported by Chinese scholars Yang Rui (15) and others, in addition to being influenced by the late initiation of puberty in males, it may also be related to the higher latitude, long winter, and lower temperature, which reduces the outdoor activities of children and adolescents. In the female group, the mean values of bone age in the 6-year-old, 8-year-old and 11-year-old groups were roughly in line with the chronological age, while the bone age in the 15-year-old group and before was slightly higher than the chronological age, and that after the 15-year-old group was slightly lower than the chronological age, and no significant differences were found between bone age and chronological age among age groups. This indicates that the bone age of females in the age group of 15 years and before has a slight tendency to advance, and after the age of 15 years, it gradually slows down, and the bone age is smaller than the chronological age, which is similar to the results reported by the domestic scholars, Tang Yonggang (16). Calculation results show that whether in the male or female group, the absolute value of BA-CA of children and adolescents is in the range of 0.02–0.47, no big difference, within the normal range of development, analyze the reason, The region is located in the northeast plain area, for the highest latitude of our country's provincial administrative centers, the masses of people generally have a higher standard of living, with a better living habits, good living habits and nutritional level, and present a good state of bone age development. In this study, the difference between the bone age of the CHN-05 method and chronological age was close to 0, indicating that the bone age of the CHN-05 method is close to the chronological age, which is highly consistent with the results of previous reports (17). And through the linear regression analysis of bone age and chronological age, two regression equations for bone age and chronological age were proposed, y = 0.886x + 1.445 for males and y = 0.893x + 1.082 for females, with correlation coefficients of R = 0.96 for males and R = 0.95 for females, and the regression was highly significant.

In recent years, the use of artificial intelligence (AI) in various medical fields has been on the rise, particularly in radiology, due to its potential to reduce inter-observer variability and alleviate the workload of medical staff (18, 19). Spampinato, Kim, and colleagues (20, 21) developed a preliminary deep learning-based bone age assessment system using standardized radiograph databases from medical centers in North America and South Korea. Additionally, an automated bone age assessment system based on data from Chinese children and adolescents is now available (19), significantly improving the efficiency and accuracy of medical practitioners in interpreting radiographs. In this study, the CHN-05 method of bone age assessment was analyzed automatically by an AI system, and the results showed a strong correlation between the bone age values obtained and the chronological age of children. These findings suggest that AI-assisted bone-age assessment is valuable for evaluating growth and development in children from frigid-zone. With advances in medical AI, its application will expand from single image analysis to multimodal information fusion. Recently, multimodal learning frameworks including iSyncTab and iStructTab integrate imaging and tabular data through cross-modal feature interaction, structured modelling and joint representation learning, providing new strategies for medical image-aided analysis. Joint modelling of medical images and structured clinical data leverages complementary multi-source information to improve the adaptability and predictive performance of AI models in complex clinical scenarios (22, 23). These findings suggest that deep learning-based AI systems are expected to play an increasingly important role in medical practice, helping to enhance the accuracy of bone age assessment.

This study has some limitations. First, as this is a retrospective single-cohort study, the broad clinical applicability of the CHN-05 method cannot be fully verified without external independent cohort validation. Second, it may be subject to selection bias. Although the data were recalculated and validated multiple times, the sample size remains relatively small, especially in the higher age groups, which had fewer participants. Furthermore, all data samples were drawn from individuals attending routine hospital physical examinations. A more comprehensive approach to evaluating bone age development in children and adolescents would involve conducting a stratified sampling study based on schools and grade levels, which would likely yield more reliable and representative results.

## Conclusion

The CHN-05 method evaluates the bone age of children and adolescents, and there are gender differences in the comparison of male and female bone age; the proportion of children and adolescents who are advanced is higher for females than for males, and the proportion of males who are delayed is higher than that of females. Males have a slightly delayed trend in pre-puberty and puberty (before 14 years old), and bone age in late puberty (after 14 years old) slightly exceeds the chronological age, showing a trend of catching up. In females, the bone age slightly advanced in the age group of 15 years and before, and gradually slowed down after 15 years of age, and the bone age was smaller than the chronological agee. Due to the above characteristic findings and the establishment of the regression equation for predicting the chronological age, it provides a reference for the growth and development of children and adolescents and contributes to the improvement of the bone age database.

## Data Availability

The raw data supporting the conclusions of this article will be made available by the authors, without undue reservation.
